# Acid-Catalyzed Liquefaction of Biomasses from Poplar Clones for Short Rotation Coppice Cultivations

**DOI:** 10.3390/molecules27010304

**Published:** 2022-01-04

**Authors:** Ivo Paulo, Luis Costa, Abel Rodrigues, Sofia Orišková, Sandro Matos, Diogo Gonçalves, Ana Raquel Gonçalves, Luciana Silva, Salomé Vieira, João Carlos Bordado, Rui Galhano dos Santos

**Affiliations:** 1 CERENA-Centre for Natural Resources and the Environment, Instituto Superior Técnico, Av. Rovisco Pais, 1049-001 Lisboa, Portugal; ivo.paulo@tecnico.ulisboa.pt (I.P.); luis.d.costa@ist.utl.pt (L.C.); sofia.oriskova@tecnico.ulisboa.pt (S.O.); sandro.matos@tecnico.ulisboa.pt (S.M.); diogo.azevedo.goncalves@tecnico.ulisboa.pt (D.G.); raquelpgoncalves95@hotmail.com (A.R.G.); lbsilva@fc.ul.pt (L.S.); salomevieira@tecnico.ulisboa.pt (S.V.); jcbordado@ist.utl.pt (J.C.B.); 2 INIAV—Instituto Nacional de Investigação Agrária e Veterinária, I.P., Ministry of Agriculture, 2780-159 Oeiras, Portugal; abel.rodrigues@iniav.pt; 3 IDMEC—Instituto de Engenharia Mecânica, Instituto Superior Técnico, Av. Rovisco Pais, 1049-001 Lisboa, Portugal; 4 WOODCHEM SA., Estrada das Moitas Altas, 2401-902 Leiria, Portugal

**Keywords:** poplar, genotypes, liquefaction, short rotation crops

## Abstract

Liquefaction of biomass delivers a liquid bio-oil with relevant chemical and energetic applications. In this study we coupled it with short rotation coppice (SRC) intensively managed poplar cultivations aimed at biomass production while safeguarding environmental principles of soil quality and biodiversity. We carried out acid-catalyzed liquefaction, at 160 °C and atmospheric pressure, with eight poplar clones from SRC cultivations. The bio-oil yields were high, ranging between 70.7 and 81.5%. Average gains of bio-oil, by comparison of raw biomasses, in elementary carbon and hydrogen and high heating, were 25.6, 67, and 74%, respectively. Loss of oxygen and O/C ratios averaged 38 and 51%, respectively. Amounts of elementary carbon, oxygen, and hydrogen in bio-oil were 65, 26, and 8.7%, and HHV averaged 30.5 MJkg^−1^. Correlation analysis showed the interrelation between elementary carbon with HHV in bio-oil or with oxygen loss. Overall, from 55 correlations, 21 significant and high correlations among a set of 11 variables were found. Among the most relevant ones, the percentage of elementary carbon presented five significant correlations with the percentage of O (−0.980), percentage of C gain (0.902), percentage of O loss (0.973), HHV gain (0.917), and O/C loss (0.943). The amount of carbon is directly correlated with the amount of oxygen, conversely, the decrease in oxygen content increases the elementary carbon and hydrogen concentration, which leads to an improvement in HHV. HHV gain showed a strong positive dependence on the percentage of C (0.917) and percentage of C gain (0.943), while the elementary oxygen (−0.885) and its percentage of O loss (0.978) adversely affect the HHV gain. Consequently, the O/C loss (0.970) increases the HHV positively. van Krevelen’s analysis indicated that bio-oils are chemically compatible with liquid fossil fuels. FTIR-ATR evidenced the presence of derivatives of depolymerization of lignin and cellulose in raw biomasses in bio-oil. TGA/DTG confirmed the bio-oil burning aptitude by the high average 53% mass loss of volatiles associated with lowered peaking decomposition temperatures by 100 °C than raw biomasses. Overall, this research shows the potential of bio-oil from liquefaction of SRC biomasses for the contribution of renewable energy and chemical deliverables, and thereby, to a greener global economy.

## 1. Introduction

As society and scientific knowledge develop, it becomes increasingly important to reduce the dependency on petrochemicals due to their dwindling reserves and their negative impact on the environment inflicted by their exploration. The European Union (EU) has set a goal to increase the budget for R&D into eco-innovations due to the importance that the European Commission has identified in this field [1], with a 294.5 billion € investment in 2020, corresponding to about 2.18% of GDP [2]. With this, the EU wants to take a leading role in the development of policies that aim to propel industries and practices into a more sustainable and environmentally friendly future.

Lignocellulosic biomasses are a renewable, continuous, and sustainable feedstock delivering liquid, gaseous, and solid materials useful for several industries [3,4]. Some significant hurdles prevail in their competitiveness by comparison with petrochemical sources. The costs of harvesting, manufacturing lines, the environmental management, the logistics of transport of light lignocellulosic materials, and their conversion wastes are examples of their drawbacks which tend to be surpassed as the need to accelerate a transition to green and circular economy gains relevance. Challenges are also posed [5] in the domain of socio–cultural–economic impacts of tree harvesting, deforestation, and the destruction of century-old forests. Given the complexity of these issues, each country identifies (within its sphere of direct influence) the most attractive lignocellulosic materials and waste streams to employ as feedstock for valorization [6].

Alternative forms of forest and land management are thus required to control the risks of excessive deforestation and the uncontrolled use of lignocellulosic materials. Short rotation coppicing comes as a possibility for biomass production while safeguarding ecosystem biodiversity and soil quality. These coppices are carbon neutral and intensively managed industrial crops, with productive cycles between two to five years, a plant density between 1200 to 10,000 per hectare, and six to seven productive cycles. The lands are subjected to fallow/rotation after that. In Europe, poplar is the predominant SRC species due to its high biomass productivity (ranging between 12 and 20 Mgha^−1^y^−1^ or higher) and its great potential for genetic improvement [7,8,9,10,11].

Poplar (*Populus* sp.) is common in SRC cultivations, with well-known fuel aptitudes [12,13] that can be hybridized to improve the bark/wood ratio, resistance to diseases, or calorific power. Besides ash and water, poplar wood contains major extractive organic compounds (ranging between 1.5 and 3%) and biopolymers, such as lignin, cellulose, and hemicellulose (ranging between 17 and 23%, 43 to 46%, and 29 to 36%, respectively) [14].

Recently published work on thermochemical conversion of poplar clones from SRCs includes studies on torrefaction [15,16], pyrolysis [17,18,19,20], and hydrothermal liquefaction [21], and solvent liquefaction [6,22,23,24,25].

Our research work focused on converting eight SRC poplar clones through acid-catalyzed liquefaction, allowing the conversion of biomass thermochemically through mild temperatures at ambient pressure [26]. A bio-oil with a high heating value greater than 40 MJkg^−1^ was previously obtained through this process [27].

Thermochemical liquefaction of different biomass feedstocks has been studied, including spruce [28], pinewood [22,29,30], eucalyptus [31,32,33], potato peels [34], cork powder [35], spent coffee beans [36], beech [37], or wheat straw [28,38,39]. The results from such studies demonstrated that different biomasses, with distinct chemical compositions and structures, can be used for the acid-catalyzed liquefaction to produce bio-oils in high yields. The previous studies demonstrated, the produced liquid bio-oil can be further used as an environmentally friendly raw material for the chemical industry or fuels [18,26,40,41,42,43,44,45,46].

The acid-catalyzed liquefaction is a thermochemical process that converts the main biopolymers (cellulose, hemicelluloses, and lignin) into low molecular weight compounds, a bio-oil. The properties of the obtained bio-oil are close to those of petroleum except for the oxygen content. For instance, from the soybean’s liquefaction, a bio-oil with a higher heating value of 44.22 MJkg^−1^ and a H/C molar ratio of 1.9 was obtained [27].

For this work, we chose eight samples of different SRC poplar genotypes as the feedstock for the acid-catalyzed liquefaction process based on our experience with this species. Poplar proliferates preferably in milder climates, such as those found in southern Europe, even though some plantations produce satisfactory yields in northern Europe as well [47]. Given the potential of SRC for biomass production, under environmental sustainability, the proposed objectives target enlarging the scope of thermochemical conversion for added-value products, e.g., chemicals and fuels. The acid-catalyzed liquefaction was applied to an array of commercial poplar clones, to study their potential to produce bio-oil in high yield. We characterized the bio-oils and solid residues to assess their use as biofuels and chemicals. To the best of our knowledge, the comparison between liquified biomasses from different poplar clones was never disclosed. Within this context, our work supports further development on thermochemical conversion technologies to value this type of biomass feedstock.

## 2. Materials and Methods

We employed eight different poplar genotypes as biomass feedstocks. Their genotype, origin, parentage, and hemicellulose, cellulose, and lignin content are shown in Table 1. The selected genotypes were *AF8*, *Bakan* (Bak), *Brandaris* (Bra), *Ellert* (Ell), *Grimminge* (Gri), *Hees* (Hee), *Skado* (Ska), and *Wolterson* (Wol). The biomass samples were not pre-treated, except from shredding on a Retsch© SM 2000 mill equipped with a 4 mm sieve to decrease the grain size and thus increase the surface area. We purchased the solvent 2-Ethylhexanol and the catalyst 97% p-Toluenesulfonic acid (PTSA) from Sigma–Aldrich. Technical acetone for washing purposes was acquired locally.

### 2.1. Liquefaction Procedure

The liquefaction, an acid-catalyzed process, was performed at 160 °C, at ambient pressure, for the predetermined reaction time. The biomass samples and the solvent were fed into the LENZ Glass Reactor, in a solvent:biomass ratio of 5:1. 2-Ethylhexanol (2-EH) was used as a solvent and the weight of biomass was based on its dry state.

The mass of catalyst, PTSA, was set at 3% (*w*/*w*) of the mass of solvent and biomass samples. After 90 min at 160 °C, the process was quenched to 80 °C. Afterward, the bio-oil crude was filtered to retrieve the solid residues.

Upon process completion, the reactor cooled to room temperature to be further vacuum filtrated. The solid residues were washed with acetone and dried in the oven at 110 ± 3 °C for 24 h. The excess solvent of the bio-oil samples was removed under a vacuum. The process conversion, bio-oil yield, was calculated as per the weight of the solid fraction obtained after filtration, according to Equation (1):

Bio-oil yield (%) = (1 − m_s0_/m_si_) × 100, (1)

where m_si_ is the mass of dry biomass fed to the reactor, in grams, and m_s0_ is the mass of solid residues obtained at the end of the process, in grams.

### 2.2. Fourier Transformed Infrared (FTIR-ATR) Analysis of Biomass and Bio-Oil

The FTIR-ATR analysis was performed on a Spectrum Two–Perkin Elmer spectrometer. The spectra were captured from 4000 to 600 cm^−1^ and treated in Perkin Elmer–Spectrum IR software.

### 2.3. Elemental Analysis

The carbon (C), hydrogen (H), and nitrogen (N) content in biomass, solid residues, and bio-oil were assessed by a LECO TruSpec CHN analyzer, whilst a LECO CNS2000 analyzer determined sulfur (S) content.

### 2.4. Higher Heating Value (HHV) Calculation

Commonly, biomass and its derivatives, i.e., bio-oil and residues, contain up to 97–99% of C, H, O. Additional elements, such as sulfur and nitrogen, are present in negligible amounts, below the detection limit, and thus difficult to measure or quantify [22,49]. We assessed the oxygen content according to Equation (2):

O (%) = 100 − C (%) − H (%),(2)

According to Rodrigues et al. [15], the elemental analysis of poplar clones vary from 51.5–52.2%, 5.2–5.4%, 42.0–43.3%, and 0.4–0.7% for C, H, O, and N, respectively. The elemental composition of poplar clones in SRC in the Czech Republic was similar to such values [13].

The higher heat value (HHV) of the biomass and solid residues was assessed using the method disclosed by Yin et al. [50] using Equation (3). The HHV of bio-oils was evaluated by Equation (4), which is specifically established for bio-oils [51].

HHV (MJ/kg) = 0.2949C + 0.8250H,(3)

HHV (MJ/kg) = 0.363302C + 1.087033H − 0.1009920,(4)

### 2.5. Energy Densification Ratio (EDR) Calculation

The energy densification ratio (EDR), a dimensionless indicator, informs on how the HHV was improved thanks to the liquefaction process [52]. We used Equation (5) to calculate the EDR values:

EDR = HHV_bio-oil_/HHV_biomass_, (5)

where HHV_bio-oil_ and HHV_biomass_ are the higher heating values of bio-oil and biomass samples, respectively.

### 2.6. Van Krevelen Diagram

The van Krevelen diagrams are useful to spot variations between different types of kerogen and fuels. This diagram cross-plots the hydrogen and carbon atomic ratio (10H/C) as a function of the oxygen to carbon atomic ratios of carbonaceous compounds. The van Krevelen diagram is suitable for identifying and revealing compositional differences between organic products [45]. Using the data obtained via elemental analysis, we plotted the chemical compositions of biomass, bio-oil, and solid residues in the van Krevelen diagram.

### 2.7. Thermogravimetric Analysis (TGA)

The thermogravimetric analysis of raw biomass, bio-oils, and solid residues was performed using the Hitachi-STA7200. The evaluation was accomplished between 25 and 600 °C in a nitrogen atmosphere, with a 100 mL/min flow and a heating rate of 5 °C/min.

### 2.8. Pearson’s Correlations

The ultimate analysis and HHV data were used to access correlations between 11 variables using SPSS Statistics software. The analysis was performed to find a correlation pattern within the bio-oil variables and quantify the interactions between them. The C, H, and O content of the bio-oils were correlated with the gain of C, H, H/C, O/C, HHV, and with the loss of O, ash, and moisture content. The number of correlations was assessed according to Equation (6):

N Pearson’s r = (n^2^ − n)/2, (6)

where N is the number of correlations and n is the number of variables.

## 3. Results and Discussion

Poplars are increasingly used for biofuel production due to their high growth and biomass productivity. They have a significant holocellulose (ranging between 47 and 52% for cellulose and 19 and 24% for hemicelluloses) and a moderate lignin content (ranging between 26 and 30%). This work presents the liquefaction results of eight different poplar genotypes. The experimental conditions, such as solvent and catalyst, were previously optimized in studies for other biomasses [29,32,33], as well as for poplar [26]. In particular, the system 2-ethylhexanol/PTSA has been shown to allow the production of bio-oils in high yields [22,53].

**Figure 1:**
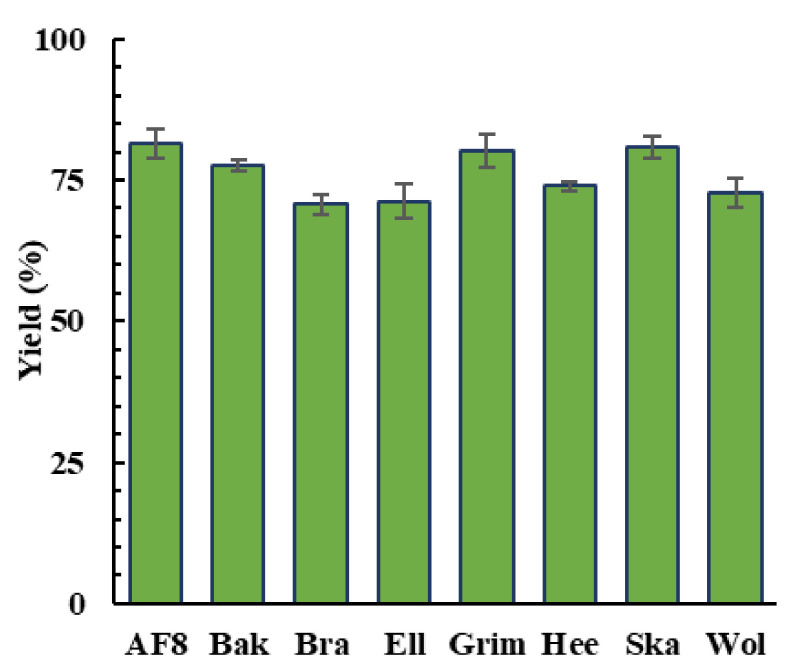
Comparison of the yield of bio-oils obtained via acid-catalyzed liquefaction of poplar clones.

For comparison, results from Rodrigues et al. [15] regarding poplar genotypes’ torrefaction were used. The liquefaction assays were conducted in duplicate at 160 °C for 90 min, using 3 wt. % PTSA as the catalyst and 1:5 biomass:solvent ratio. At first glance, bio-oil yields higher than 70% indicated the SRC poplar clones’ aptitude for liquefaction. The bio-oil yields are shown in Figure 1. Overall, the process led to a bio-oil yield ranging from 70.7 to 81.5%. The highest bio-oil yields of around 81% were obtained for the *AF8* and *Skado* genotypes, while the lowest conversion was achieved with the *Brandaris* sample. Overall, the conversions are in accordance with the literature concerning similar thermochemical conversion under the same experimental conditions [29,54]. In comparison, the microwave-assisted pyrolysis of poplar, where the bio-oil achieved a maximum of 30.8%, and the conducted liquefaction process led to higher yields [17].

It should be noted that the solid residue fraction can be given by the difference for the complete conversion once the gaseous streams are reduced and can be neglected [29,30]. The solids contain unreacted biomass as well as any decomposition products. In fact, during the liquefaction, solid residues can be produced from the decomposition of lignocellulosic biomass, and are commonly referred to as humins [29,30,33]. Such occurrence is well-explained and is associated with the recondensation of decomposition products of reactions [55,56,57].

The chemical characterization of biomass and its bio-oil counterparts, as well as the solid residues and torrefied samples, is shown in Table 2. It encompasses the ultimate analysis, moisture, and ash content, calculated HHV, H/C, and O/C ratios. Table 3 presents the values of % C gain, % H gain, % O gain, % ash loss, % moisture loss, H/C gain, and O/C loss of the bio-oils from biomass liquefaction, compared to the untreated biomass samples.

**Figure 2:**
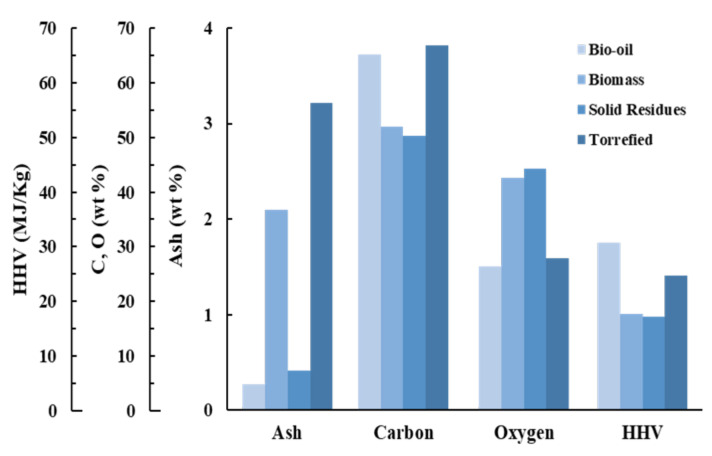
Comparison of the average ash, carbon, oxygen contents, and HHV between bio-oils, biomass, solid residues, and torrefied biomass.

The bio-oil elemental analysis is in accordance with those obtained for other biomasses, e.g., pinewood, eucalyptus, and tomato pomace [22,30,32,58]. The results from the chemical analysis of bio-oil proved that the sets of liquefied biomasses were very distinct from the chemical composition of the raw biomasses and the solid residue. The carbon content (%, dry basis) was higher for the bio-oil, ranging from 64.4% (*AF8* and *Brandaris*) to 66.1% (*Ellert*). On the other hand, the elementary carbon content of the residues averaged 50.19%, presenting values between 48.6% (*Hees*) and 52.3% (*AF8*). While the solid residues showed a slightly lower elementary carbon content than the feedstock, the% carbon gain for the bio-oils was, on average, ~25%. An advantage of bio-oil is that its ash content is much lower than that of the torrefied biomass. The liquefaction delivered bio-oils with a very similar average carbon content (~67%) to that from the torrefied biomass (see Figure 2) [15].

Regarding hydrogen content (%, dry basis), the bio-oils, averaging ~8.7%, presented values ranging from 8.4% (*AF8* and *Skado*) to 8.9% (*Bakan, Grimminge, Wolterson*) (Table 1). Such values demonstrated an average 65% gain in the hydrogen content by comparison with the raw biomass. The solid residues presented a hydrogen content like that from the raw biomass. Additionally, the hydrogen content was up to 75% higher than the torrefied biomass, wherein losses of hydrogen and H/C ratios were detected [15].

As expected, the oxygen content (%, dry basis) was considerably lower for the bio-oils and torrefied biomass compared to their biomass counterparts, with values averaging ~26%, ~28%, and ~42%, respectively (see Figure 2). The highest oxygen content was obtained for the genotype *AF8* genotype samples (27.2%) and the lowest for genotype *Ellert* (25.1%). The correspondent oxygen amount of the solid residues were in line with those of the raw biomasses. The average % O loss (~38%) was concomitant with increases in C and H contents of around 25.61% and 65.17% (Table 2). The loss of oxygen occurs through water elimination, which is retrieved by distillation during the process [29]. Consequently, the H/C and O/C ratios showed significant gains of around 32 and 51% (Table 2). The ash and moisture content ranged from 0.1 to 0.4% and 0.96 to 1.49%, respectively, for the obtained bio-oils. A significant decrease in ash and moisture content compared with the raw poplar genotypes was obtained upon biomass liquefaction (see Figure 2).

The average ratios O/C and 10H/C of bio-oils from biomass poplar clones were 0.40 and 1.34, respectively (Table 1). The variations of these ranges between clones were small, with the O/C ratio ranging between 0.38 and 0.42 and the 10H/C ratio ranging between 1.31 and 1.38. The O/C of bio-oils was lower than that of raw poplar biomass samples and like the O/C values of torrefied poplar clones. On the other hand, the H/C increased considerably due to the rise in hydrogen in bio-oil samples.

These variations explain the improvement in the calculated HHV since lower oxygen content and O/C ratios lead to higher HHV. On average, the bio-oils presented an HHV of 30.49 MJ/kg. *AF8* presented the lowest value (29.85 MJ/kg), while *Wolterson* presented the highest (30.84 MJ/kg). The HHV of the biomass was on average 17.51 MJ/kg, and that of torrefied biomass was 24.5 MJ/kg, demonstrating that the HHV was remarkably improved with the liquefaction. The presence of residual solvent can also potentiate, although not to the fullest extent, the observed increase on the HHV. However, the solvent was removed, and work from Condeço et al., 2021, showed that liquefaction processes with low conversion led to lower HHV oil, indicating that increases in HHV result from the conversion of the lignocellulosic materials into bio-oil [59]. The values of Ma et al. [27], although higher (>40 MJkg^−1^) than those obtained in this work, were indicative of the potential of this solvolytic liquefaction for delivering bio-oil with high heating power.

The HHV gain of bio-oil by comparison with raw biomass averaged 74%. Compared with torrefied biomass [15], wherein the increase in HHV was ~40%, bio-oil still presented a high energy densification ratio (74%). Figure 2 highlights the significant increase in the % elemental carbon and the decrease in the % O content, which increases the HHV of the biomass when compared to the raw and torrefied biomass.

The energy densification ratio (EDR) was employed to calculate the effectiveness of the process. The increase in the EDR results from solid mass decrease due to dehydration and decarboxylation reactions [52]. The average bio-oil EDR of 1.74 showed that the liquefaction of poplar biomass led to higher energy densification. On the other hand, the solid residues led to a slight loss in the heating values, which accounted for an EDR of 0.97. The lower average EDR of 1.39 for torrefied biomass reflected the aptitude of liquefaction for delivering a bio-oil product with high calorific potential.

The van Krevelen diagram identified the fuel quality changed with the chemical composition variation. Usually, biomasses with lower O/C and H/C ratios are considered good fuel aptitudes due to lower water vapor, minimum energy loss, and less smoke upon combustion [60].

**Figure 3:**
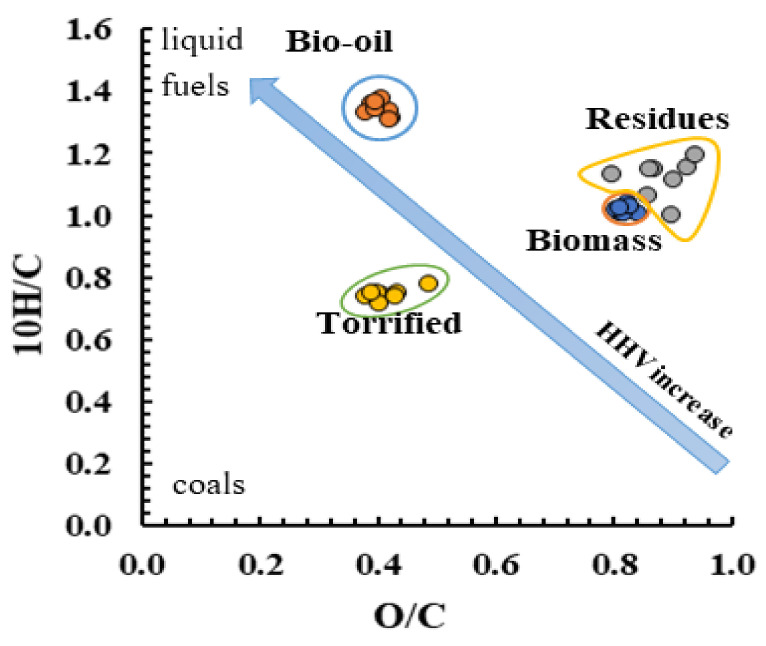
Van Krevelen diagram comparing the H/C and O/C ratios of biomass, solid residues, torrefied biomass, and bio-oils.

Overall, the van Krevelen diagram (Figure 3) showed that the bio-oil locations were close to those of liquid fossil fuels (such as diesel or gasoline), demonstrating that liquefaction leads to liquid products similar to fossil fuels. By comparison, torrefaction leads to products similar and compatible with fossil coals/peat. On the other hand, the solid residues were closer to the highly oxidized compounds. In comparison with biomass, the atomic ratios of O/C and H/C of solid residues increased, while for bio-oil, the H/C increased, and O/C decreased. This suggests that bio-oil is a better fuel than raw biomass itself. The decrease in O/C atomic ratios leads to an increase in the high energy bonds (C-C) and a reduction in low energy bonds (O-C) leading to an HHV improvement. González-Arias et al. postulated that such change might be explained by the occurrence of dehydration reactions that leads to hydroxyl groups loss and by the decarboxylation reactions that eliminate the carboxyl and carbonyl groups [52].

The distribution of Pearson correlations among chemical variables and calorific power of bio-oils and raw biomass samples reflected the above-described tendencies (Table 4). From 55 correlations, 21 significant and high correlations (r > 0.7) among a set of 11 variables were found. Among the most relevant, the % elementary carbon presented five significant correlations with % O (−0.980), % C gain (0.902), % O loss (0.973), HHV gain (0.917), and O/C loss (0.943). As expected, the amount of carbon was directly correlated with the amount of oxygen. The decrease in oxygen content increased the elementary carbon and hydrogen concentration, which led to an improvement in HHV. The HHV gain showed a strong positive dependence with the % C (0.917) and % C gain (0.943). Conversely, the elementary oxygen (−0.885) and its % O loss (0.978) adversely affected the HHV gain. Consequently, the O/C loss (0.970) increased the HHV positively.

**Figure 4:**
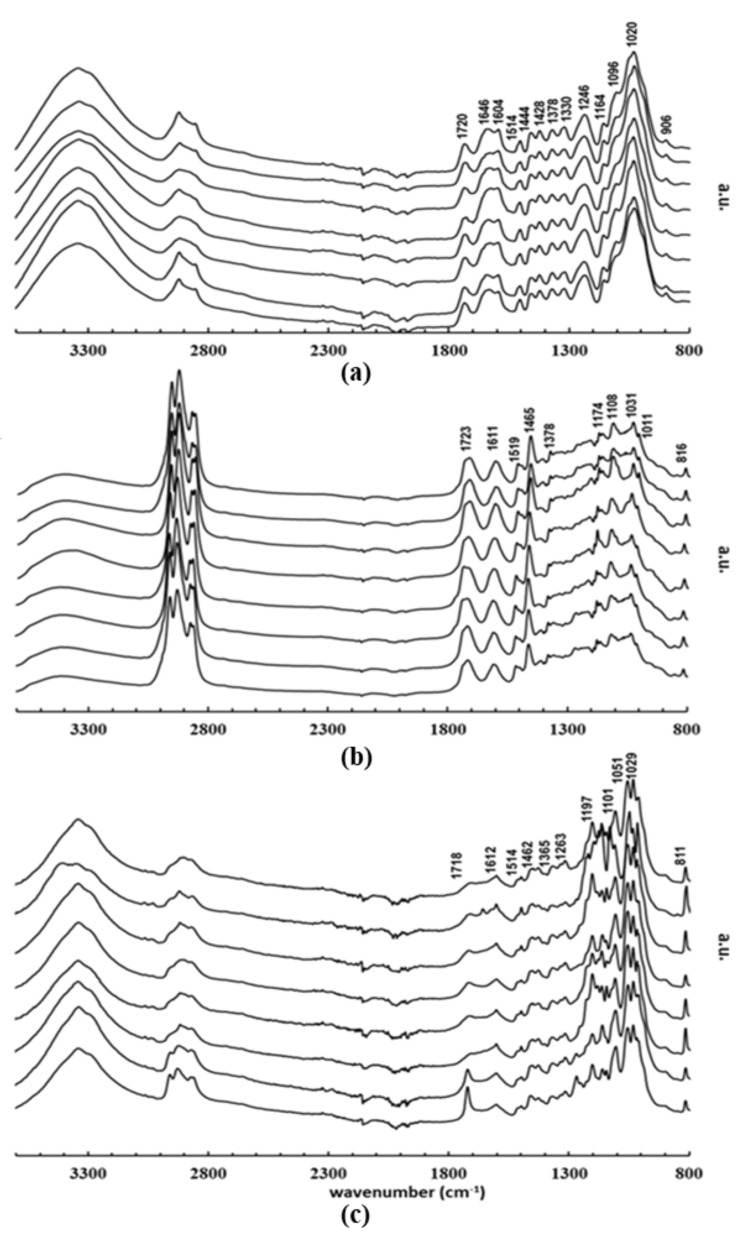
FTIR-ATR spectra of (**a**) biomass; (**b**) bio-oil, and (**c**) solid residue.

Figure 4 and Table 5 show the ATR-FTIR spectra and data of raw biomass (Figure 4a), bio-oil (Figure 4b), and solid residues (Figure 4c) for the eight poplar genotypes. No significant differences in the profiles of functional groups were detected among samples of poplar genotypes within each profile. The major spectral differences concerned the absorption intensity in the range between 2800 and 3000 cm^−1^ assigned to C–H stretching vibrations. In this range, the bio-oils had a higher absorption than biomass and solid residues. In the range 1370 and 1730 cm^−1^, solid residues have visibly lower absorption than bio-oils or biomass. In the range 1100–1200 cm^−1^ we saw lower absorption in bio-oils in comparison with biomass and solid residue samples. Overall, the spectra of all samples displayed a broad band around 3500 cm^−1^, a characteristic band resulting from OH stretching vibration.

The absorption differences between 2800 and 3000 cm^−1^, assigned to C–H stretching vibrations, point out the presence of derivatives of holocellulose and lignin in bio-oil. In the range from 1370 to 1730 cm^−1^, biomass spectra showed the peaks at 1604 and 1514 cm^−1^, generally attributed to the presence of lignin. These same peaks were identified on the bio-oil spectra at 1611 and 1519 cm^−1^, respectively, and were practically non-existent in the spectra of solid residues. These peaks in bio-oil spectra revealed that lignin was depolymerized, hence its derivatives were present. Moreover, the peaks related to syringyl and guaicyl units at 1378 and 1246 cm^−1^, respectively, were identified within the bio-oil samples. On the other hand, the peaks related to hemicellulose and cellulose associated with the stretching and vibrations of functional groups (see Table 5) were identified in the biomass as well as the bio-oil samples (peaks at 1465, 1174, 1108, 1031 cm^−1^). The differences in absorption in the range from 1100 to 1200 cm^−1^ reflected the inherent chemical differences between the profile of bio-oil and the other two. Additionally, at 1723 cm^−1^, a peak was shown due to the vibrational states of carbonyl functional groups present in aldehydes, ketones, acids, or esters, which resulted from the conversion of cellulose or hemicellulose into levulinic acid, furfural, and related compounds [61]. On the other hand, in the biomass, the correspondent peak profiled in the biomass sample spectra (1720 cm^−1^) is associated with hemicellulose and lignin [59]. The peak at 1646 cm^−1^, assigned to the OH bending of water, confirmed the presence of water in the biomass samples. Regarding the solid residues’ spectra, peaks related to lignin were identified at 1612, 1514, 1365, and 1263 cm^−1,^ and those concerning holocellulose appeared at 1462, 1197, 1101, and 1029 cm^−1^ (Table 5). These peaks indicated the presence of unreacted biomass in solid residues.

**Figure 5:**
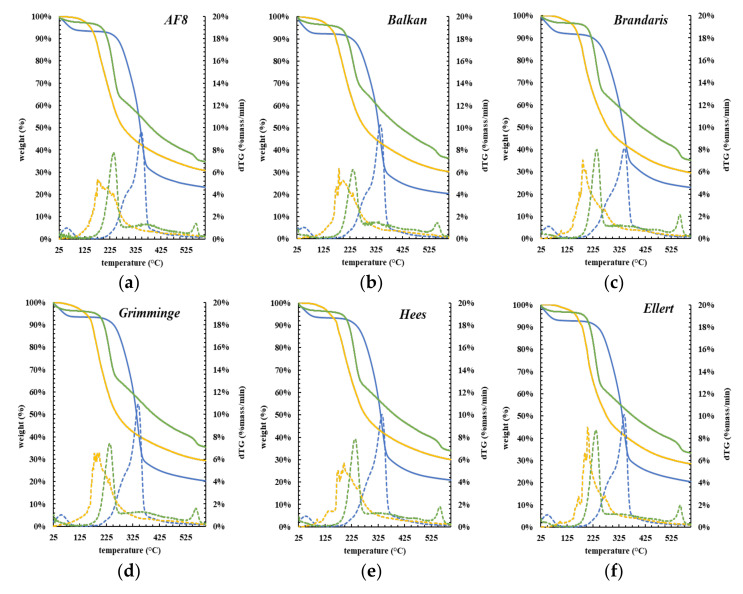

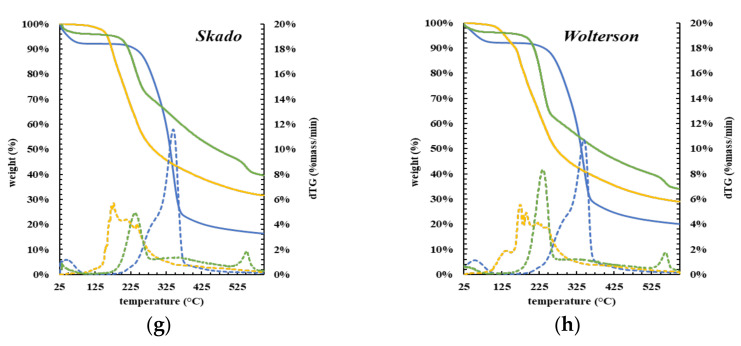
TGA and DTG thermograms of biomass (blue), bio-oil (yellow), and solid residues (green) of all poplar clones: (**a**) *AF8*; (**b**) *Balkan*; (**c**) *Brandaris*; (**d**) *Grimminge*; (**e**) *Hees*; (**f**) *Ellert*; (**g**) *Skado*; (**h**) *Wolterson*. The dashed line is gTG and the solid line is TGA.

The TGA/DTG curves and mass losses of biomass, bio-oils, and solid residues between 0 and 600 °C, are shown in Figure 5 and Table 6. Regarding raw biomass, TGA analysis evidenced four stages of decomposition. During the first stage, at temperatures ranged between 25 °C and 120 °C, a mass loss averaging 7% occurred, concerning volatile components and free water content. The second (120–300 °C) and third stages (300–400 °C) averaged weight losses of 19 and 47%, respectively. The biomass’s biopolymolecular structure suffered restructuration at this point, releasing smaller compounds (e.g., H_2_O, CO, CO_2_, etc.). The cellulose and hemicellulose, alongside lignin, decomposed to form volatiles and low molecular weight compounds during these two stages. While the decomposition of holocellulose led mainly to volatiles; lignin produces primarily carbon. Cellulose, xylan, and lignin contained about 91, 77, and 66% of volatile matter, respectively. The fourth stage, beginning at 400 °C, involved a slower decomposition and significantly lower mass loss of 7% (similar to the first stage) and was associated with the volatilization of carbon via C–C and C–H bonds cleavage [71].

The TGA curves analysis showed that bio-oils from poplar liquefaction were more volatile than the fresh raw material, thus requiring lower peaking temperatures to vaporize and decompose. The maximum temperatures of TGA decomposition were about 325 °C and 225 °C for raw biomasses and bio-oil, respectively. The TGA curves also revealed that the bio-oils decomposed in a three-stage pattern, between about 50 °C and 600 °C (Table 6). The onset temperature of thermal decomposition of bio-oils was about 50 °C. The bio-oil samples presented the first weight loss, ca. 16%, up to 185 °C, due to volatilization of moisture and low molecular weight compounds. From 180–300 °C, the second stage exhibited an average mass loss of ~37%, corresponding to the bio-oil’s heavier components that require low temperatures to decompose or volatilize. Seehar et al. hypothesized that the mass loss at these temperatures might denote the presence of chemical structures analogs to those from gasoline, diesel, and jet fuel [72]. The third stage (300–600 °C) showed an average mass loss of 17%, which can be attributed to residual char formation from the sample’s slow degradation.

Generally, the thermogravimetric curves of bio-oil samples showed an average mass loss of up to 70%. The mass loss as volatiles up to 300 °C, summing up ca. 53%, can denote some gasoline, jet fuel, and diesel segments [72]. Such decomposition profile supports their potential use in combustion applications [73]. The DTG curves showed that the bio-oil dropped weight at lower temperatures, confirming the presence of a lighter product than their biomass counterparts. Most of the mass loss was verified below 230 °C.

The TGA curves of the solid residues showed four decomposition stages. Within the first stage (temperatures up to 115 °C) they showed a low average mass loss of 3%, related to the loss of moisture and other light compounds. The second (125–260 °C) and third (260–525 °C) stage of thermal decomposition of solid residues corresponded to average mass losses of 30 and 26%, respectively. Such stages displayed peaking temperatures of 325 °C and 250 °C, typical of cellulose and hemicellulose, respectively, suggesting that the biomass liquefaction was incomplete [59]. In the fourth stage, corresponding to temperatures higher than 525 °C, a slight mass loss of 6% was attributed to heavy compounds resulting from the condensation of liquefaction products that led to insoluble solids. It is worth noting that, on DTG curves, a slight mass loss above 350 ºC, <2%mass/min, led to the peak of the 4th stage. This suggests the presence of heavy compounds by comparison with the DTG of biomass and bio-oils.

## 4. Conclusions

This study evaluated the aptitude of bio-oils obtained via acid-catalyzed liquefaction of poplar woody biomasses from eight clones from short rotation crops. The laboratory assays were performed under mild conditions of 160 °C and ambient pressure, and the resulting bio-oil yield ranged between 70.7 and 81.5%, within the scope of cited literature. Loss of oxygen and O/C ratios averaged 38 and 51%, respectively. Elementary amounts of carbon, oxygen, and hydrogen in bio-oil were 65, 26, and 8.7%, respectively, and HHV averaged a value of 30.5 MJkg^−1^. Correlation analysis showed the interconnectedness between, e.g., elementary carbon with HHV in bio-oil or with oxygen loss. The van Krevelen diagram proved that bio-oils are more chemically compatible with liquid fossil fuels, such as diesel or gasoline than the initial biomass. FTIR analysis evidenced the drastic chemical conversion of raw woody biomass through the presence of derivatives of depolymerization of lignin and holocellulose in bio-oil. Results of TGA/DTG in a nitrogen atmosphere confirmed the burning aptitude of bio-oil by the high mass losses of volatiles of 53% and by peaking decomposition temperatures lowered by 100 °C than those of raw biomasses. Overall, the TGA analysis showed that bio-oils from poplar liquefaction were more volatile than the fresh feedstock, thus requiring lower peaking temperatures to vaporize and decompose. Additionally, in comparison with biomass, the bio-oil atomic ratios of H/C increased, and O/C decreased. This reflects the fact that bio-oil is a better fuel than raw biomass. Liquefaction results from this research confirmed the potential of biomasses from SRC cultivations to produce energy and chemicals.

## Figures and Tables

**Table 1:** Hemicellulose, cellulose, and lignin estimated the content of the poplar genotypes [48].

			Lignocellulosic Content (%)		
Genotype	Origin	Parentage	Hemicellulose	Cellulose	Lignin
*AF8*	Portugal	Hybrid P. generosa	23	48	28
*Bakan*	Belgium	Hybrid P. trichocarpa × P. maximowiczii	19	52	28
*Brandaris*	Belgium	Species P. nigra	23	47	29
*Ellert*	Belgium	Hybrid P. canadensis	24	48	26
*Grimminge*	Belgium	Triple hybrid P. deltoides × (P. trichocarpa × P. deltoides)	24	48	27
*Hees*	Belgium	Hybrid P. canadensis	23	50	26
*Skado*	Belgium	Hybrid P. trichocarpa × P. maximowiczii	20	49	30
*Wolterson*	Belgium	Species P. nigra	24	48	27

**Table 2:** Chemical characterization of poplar clones, bio-oils, solid residues, and torrefied biomass.

	Samples	Chemical Composition ^1^ (%)				Ash (%)	Moisture (%)	HHV ^2^ (MJ/kg)	10H/C	O/C	Empirical Formula
		C	H	N	O						
Biomass [15]	*AF8*	51.5	5.2	<0.5	43.3	2.18	9.58	17.20	1.01	0.84	CH_1.22_O_0.61_
	*Bakan*	51.5	5.4	0.6	42.5	1.56	10.80	17.49	1.04	0.83	
	*Brandaris*	52.0	5.2	0.7	42.1	2.87	8.08	17.58	1.00	0.81	
	*Ellert*	51.8	5.3	0.6	42.4	2.25	10.10	17.52	1.02	0.82	
	*Grimminge*	52.2	5.3	0.5	42.0	1.76	9.42	17.75	1.02	0.81	
	*Hees*	51.8	5.2	0.7	42.3	2.39	7.90	17.48	1.01	0.82	
	*Skado*	51.6	5.3	0.4	42.7	1.47	9.91	17.45	1.03	0.83	
	*Wolterson*	51.9	5.3	0.7	42.1	2.28	9.73	17.64	1.02	0.81	
	Mean	51.79	5.27	0.59	42.42	2.09	9.44	17.51	1.02	0.82	
Bio-oil	*AF8*	64.4	8.4	<0.5	27.2	0.4	1.30	29.85	1.31	0.42	CH_1.61_O_0.30_
	*Bakan*	65.6	8.9	<0.5	25.5	0.3	1.13	30.95	1.36	0.39	
	*Brandaris*	64.4	8.6	<0.5	27.0	0.3	1.49	30.03	1.33	0.42	
	*Ellert*	66.1	8.8	<0.5	25.1	0.4	1.15	31.06	1.33	0.38	
	*Grimminge*	64.8	8.9	<0.5	26.3	0.1	0.96	30.62	1.38	0.41	
	*Hees*	65.3	8.8	<0.5	25.9	0.2	1.18	30.67	1.34	0.40	
	*Skado*	64.5	8.4	<0.5	27.1	0.3	1.37	29.90	1.31	0.42	
	*Wolterson*	65.3	8.9	<0.5	25.8	0.2	1.42	30.84	1.37	0.39	
	Mean	65.05	8.72	--	26.23	0.28	1.25	30.49	1.34	0.40	
Solid residues	*AF8*	52.3	5.9	<0.5	41.8	0.3	--	18.44	1.13	0.80	CH_1.34_O_0.66_
	*Bakan*	50.4	5.8	<0.5	43.8	0.3	--	17.36	1.15	0.87	
	*Brandaris*	50.0	5.0	<0.5	45.0	0.2	--	16.25	1.00	0.90	
	*Ellert*	49.0	5.7	<0.5	45.3	0.7	--	16.52	1.16	0.93	
	*Grimminge*	50.6	5.8	<0.5	43.6	0.3	--	17.48	1.15	0.86	
	*Hees*	48.6	5.8	<0.5	45.6	0.8	--	16.48	1.19	0.94	
	*Skado*	50.9	5.4	<0.5	43.7	0.1	--	17.14	1.06	0.86	
	*Wolterson*	49.7	5.6	0.5	44.2	0.6	--	16.81	1.12	0.89	
	Mean	50.19	5.62	0.52	44.13	0.41	--	17.06	1.12	0.88	
Torrefied biomass [15]	*AF8*	66.3	4.9	0.36	28.44	3.46	--	24.2	0.74	0.43	CH_0.89_O_0.32_
	*Bakan*	65.9	4.94	0.66	28.5	2.7	--	24.1	0.75	0.43	
	*Brandaris*	66.9	4.99	0.88	27.29	4.0	--	24.6	0.75	0.41	
	*Ellert*	67.8	5.1	0.73	26.37	3.49	--	25.2	0.75	0.39	
	*Grimminge*	68.3	5.06	0.69	25.94	2.97	--	25.4	0.74	0.38	
	*Hees*	67.3	4.8	0.84	27.06	3.54	--	24.6	0.71	0.40	
	*Skado*	67.4	5.04	0.54	27.02	2.63	--	24.9	0.75	0.40	
	*Wolterson*	63.5	4.95	0.7	30.85	2.93	--	22.9	0.74	0.49	
	Mean	66.7	4.97	0.68	27.68	3.22	--	24.47	0.75	0.42	

^1^ Dry basis; ^2^ calculated HHV.

**Table 3:** Ratios of C, H, HHV H/C gain and of O, Ash, Moisture, and O/C loss of the bio-oils obtained from the liquefaction of poplar clone samples.

Sample	C Gain (%)	H Gain (%)	O Loss (%)	Ash Loss (%)	HHV Gain (%)	Moisture Loss (%)	H/C Gain (%)	O/C Loss (%)
*AF8*	25.05	61.54	37.18	81.65	73.56	95.82	29.70	50.00
*Bakan*	27.38	64.81	40.00	80.77	76.98	97.22	30.77	53.01
*Brandaris*	23.85	65.38	35.87	89.55	70.82	96.29	33.00	48.15
*Ellert*	27.61	66.04	40.80	82.22	77.26	96.04	30.39	53.66
*Grimminge*	24.14	67.92	37.38	94.32	72.50	98.94	35.29	49.38
*Hees*	26.06	69.23	38.77	91.63	75.48	97.47	32.67	51.22
*Skado*	25.00	58.49	36.53	79.59	71.34	96.97	27.18	49.40
*Wolterson*	25.82	67.92	38.72	91.23	74.83	97.94	34.31	51.85
Mean	25.61	65.17	38.16	86.37	74.10	97.09	31.67	50.83

**Table 4:** Pearson’s correlation (r) from elemental analysis and bio-oil variables.

		Variables (%)										
		C	H	O	C Gain	H Gain	O Loss	Ash Loss	HHV Gain	Moisture Loss	H/C Gain	O/C Loss
**Variables (%)**	C	1	0.669	−0.980 ^2^	0.902 ^2^	0.485	0.973 ^2^	0.042	0.917 ^2^	0.049	0.090	0.943
	H	0.669	1	−0.803 ^1^	0.389	0.814 ^1^	0.617	0.595	0.561	0.676	0.712	0.537
	O	−0.980 ^2^	−0.803 ^1^	1	−0.827 ^1^	−0.605	−0.945 ^2^	−0.192	−0.885 ^2^	−0.219	−0.261	−0.899
	C Gain	0.902 ^2^	0.389	−0.827 ^1^	1	0.150	0.947 ^2^	−0.311	0.943 ^2^	−0.201	−0.272	0.972
	H Gain	0.485	0.814^1^	−0.605	0.150	1	0.405	0.650	0.400	0.502	0.868	0.291
	O Loss	0.973 ^2^	0.617	−0.945 ^2^	0.947 ^2^	0.405	1	−0.119	0.978 ^2^	0.000	0.012	0.984
	Ash Loss	0.042	0.595	−0.192	−0.311	0.650	−0.119	1	−0.207	0.813	0.739	−0.205
	HHV Gain	0.917 ^2^	0.561	−0.885 ^2^	0.943 ^2^	0.400	0.978 ^2^	−0.207	1	−0.062	−0.001	0.970
	Moisture Loss	0.049	0.676	−0.219	−0.201	0.502	0.000	0.813 ^1^	−0.062	1	0.643	−0.068
	H/C Gain	0.090	0.712 ^1^	−0.261	−0.272	0.868 ^2^	0.012	0.739 ^1^	−0.001	0.643	1	−0.091
	O/C Loss	0.943 ^2^	0.537	−0.899 ^2^	0.972 ^2^	0.291	0.984^2^	−0.205	0.970 ^2^	−0.068	−0.091	1

^1^ *p* < 0.05; ^2^ *p* < 0.01.

**Table 5:** FTIR-ATR relevant peaks for biomass, bio-oil, and solid residues.

Peaks (cm^−1^)			Band Assignment		Ref.
Biomass	Bio-Oil	Residues	Functional Group	Compounds	
1720	1723	1718	C=O carbonyls in ester groups and acetyl groups in xylan	Ketones, esters, hemicellulose, and carboxylic acids and esters	[62,63,64]
1646			O-H bending	Water	[65,66]
1604	1611	1612	C=C aromatic ring vibration	Lignin	[62,67]
1514	1519	1514	C=C aromatic ring stretching	Lignin	[32,68]
1444	1465	1462	OCH_3_-, -CH_2_-, and C-H stretching	Cellulose, hemicellulose	[69]
1378	1378	1365	Aromatic C-H deformation	Syringyl rings	[63]
1330			C-O syringyl ring	Lignin	[62]
1246	1248	1263	Aromatic ring vibration	Guaicyl lignin	[62]
1164	1174	1197	C-O-C asymmetrical stretching	Cellulose, hemicellulose	[62]
1096	1108	1101	C-O-C stretching	Cellulose, hemicellulose	[64]
1020	1031	1029	C-O, C=C, and C-C-O stretching	Cellulose, hemicellulose, lignin	[62]
906			Glycosidic linkage	Cellulose, hemicellulose	[62,68]
	816	811	C-H out-of-plane	Cellulose, hemicellulose	[70]

**Table 6:** Mass loss from TGA curves for biomass, bio-oils, and solid residues.

Samples		TGA Curve							
		1st Stage		2nd Stage		3rd Stage		4th Stage	
		Temp. Range (°C)	Mass Loss (%)	Temp. Range (°C)	Mass Loss (%)	Temp. Range (°C)	Mass Loss (%)	Temp. Range (°C)	Mass Loss (%)
*AF8*	Biomass	<120	6	80–300	18	300–400	46	>400	7
	Bio-oil	50–185	18	185–300	35	300–600	16	--	--
	Residue	<115	3	125–260	32	260–525	26	>525	4
*Balkan*	Biomass	<120	8	80–300	20	300–400	46	>400	6
	Bio-oil	50–185	15	185–300	37	300–600	18	--	
	Residue	<115	4	125–260	26	260–525	28	>525	6
*Brandaris*	Biomass	<120	8	80–300	19	300–400	42	>400	8
	Bio-oil	50–185	16	185–300	37	300–600	17	--	--
	Residue	<115	3	125–260	30	260–525	25	>525	7
*Ellert*	Biomass	<120	7	80–300	20	300–400	46	>400	7
	Bio-oil	50–185	10	185–300	44	300–600	18	--	
	Residue	<115	3	125–260	34	260–525	24	>525	6
*Grimminge*	Biomass	<120	6	80–300	19	300–400	49	>400	6
	Bio-oil	50–185	17	185–300	38	300–600	16	--	--
	Residue	<115	4	125–260	29	260–525	26	>525	6
*Hees*	Biomass	<120	7	80–300	20	300–400	46	>400	6
	Bio-oil	50–185	16	185–300	38	300–600	16	--	--
	Residue	<115	4	125–260	31	260–525	25	>525	6
*Skado*	Biomass	<120	8	80–300	19	300–400	51	>400	6
	Bio-oil	50–185	17	185–300	35	300–600	17	--	--
	Residue	<115	4	125–260	22	260–525	28	>525	6
*Wolterson*	Biomass	<120	8	80–300	19	300–400	47	>400	6
	Bio-oil	50–185	20	185–300	35	300–600	16	--	--
	Residue	<115	4	125–260	33	260–525	23	>525	6
Mean	Biomass	<120	7	80–300	19	300–400	47	>400	7
	Bio-oil	50–185	16	185–300	37	300–600	17	--	--
	Residue	<115	3	125–260	30	260–525	26	>525	6

## Data Availability

Not applicable.

## Sample Availability

Samples of the compounds are not available from the authors.
